# Effects and Safety of Aqueous Extract of* Poncirus fructus* in Spinal Cord Injury with Neurogenic Bowel

**DOI:** 10.1155/2016/7154616

**Published:** 2016-09-25

**Authors:** Ji Hee Kim, Su Kyung Lee, Min Cheol Joo

**Affiliations:** ^1^Department of Rehabilitation Medicine, Wonkwang University School of Medicine, Iksan 570-749, Republic of Korea; ^2^Department of Rehabilitation Medicine, Wonkwang University School of Korean Medicine, Iksan 570-749, Republic of Korea; ^3^Institute of Wonkwang Medical Science, Wonkwang University School of Medicine, Iksan 570-749, Republic of Korea

## Abstract

*Objective*. To investigate the effects and safety of the aqueous extract of the dried, immature fruit of* Poncirus trifoliata* (L.) Raf., known as* Poncirus fructus* (PF), in spinal cord injury (SCI) patients with neurogenic bowel.* Methods*. Thirty-one SCI patients with neurogenic bowel were recruited. Patients were evaluated based on clinical information, constipation score, Bristol Stool Form Scale, stool retention score using plain abdominal radiograph, and colon transit time. PF was administered in dosages of 800 mg each prior to breakfast and lunch for 14 days.* Results*. The morphological feature of the stool before and after administration indicated a statistically significant difference from 3.52 ± 1.33 to 4.32 ± 1.44 points (*p* < 0.05). Stool retention score before and after administration of PF was represented with low significance (7.25 ± 1.60 to 6.46 ± 1.53 points) in the whole colon (*p* < 0.05), and the colon transit time was significantly shortened (57.41 ± 20.7 to 41.2 ± 25.5 hours) in terms of the whole transit time (*p* < 0.05). Side effects were observed in 7 people (28.0%) consisting of 2 people with soft stools and 5 people with diarrhea.* Conclusion*. For SCI patients, PF administration significantly improved defecation patterns, defecation retention, and colon transit time. PF could be an effective aid to improve colonic motility and constipation.

## 1. Introduction

Constipation due to a neurogenic bowel after spinal cord injury (SCI) is one of the most common complications and has been reported in 39.1% of patients with SCI [1]. SCI causes the loss of sensation, the loss of voluntary control of defecation, and a decrease in colonic motility. This leads to a delay in colon transit time and decreases colonic motility in scintigraphy [2]. A neurogenic bowel often restricts social activities and impairs the quality of life (QOL) [3].

Most SCI patients use many types of bowel management programs. Successful bowel management is multidimensional. Treatments may be multifaceted, using a mixture of strategies in regard to diet, medicine, electrical stimulation, and/or surgery [4]. Medications include oral laxatives, peristaltic stimulants, bulk forming agents, and stool softeners. Most commonly, one defecation stimulation method is used in 59.8% of cases, with two or more methods reportedly used in 12.2% of cases [5]. In a majority of cases (62.3%) the duration of defecation exceeds 16 min [5]. Therefore, it is necessary to establish a bowel management program for SCI patients containing appropriate bowel habit, diet, and medications to manage defecation.

The dried immature fruit of* Poncirus trifoliata* (L.) Raf. (Rutaceae), known as* Poncirus fructus* (PF), has been widely used as a traditional medicine in Eastern Asia, especially as an over-the-counter drug in Korea for the treatment of various gastrointestinal disorders [6]. Even with the general use of PF, the basis of its improvement of gastrointestinal motility is unclear. PF promotes intestinal transit in rodents with experimental gastrointestinal motility dysfunctions [7] and an aqueous water extract of PF accelerated the colon transit time in a mouse model with SCI [8]. Oral administration of PF aqueous extract was not shown to influence gastric emptying but did accelerate transit of intestinal contents [9].

Previous studies of the effect of PF on gastrointestinal motility have mostly involved animal models or normal adults without constipation. The usual recommended single doses of PF range widely within 2~75 g, with side effects including soft stools, diarrhea, and stomachache. But the types and severity of side effects according to PF dosage and administration are unclear.

The present study of SCI patients with neurogenic bowel was undertaken to clarify the change in colon motility and to evaluate the effectiveness and safety of oral PF. We assessed whether oral administration of PF improved the defecation pattern using plain abdominal radiography and altered the colon transit time.

## 2. Materials and Methods

### 2.1. Subjects

Of SCI patients who were admitted to the Department of Rehabilitation Medicine in Wonkwang University Hospital from January 2011 to June 2013, 31 patients were selected for this study after providing informed consent for plain abdominal radiography, for survey of defecation patterns, and for examination of colon transit time. Six individuals were excluded because of arbitrary administration of PF (*n* = 4), not recording Kolomark*™* dosing time (*n* = 1), and occurrence of side effects (*n* = 1). Overall, 25 patients were included as subjects for the study. Patients with congenital abnormality and previous surgery history in the gastrointestinal tract except single appendectomy or cholecystectomy were excluded.

### 2.2. Methods

The survey of defecation patterns, plain abdominal radiography, and colon transit time were evaluated for subjects before and after the 14 days of PF administration. The types and severity of side effects after PF dosing were investigated. Food intake, water intake, and rehabilitation treatment type and time were uniformly maintained during this study, and medication changes were minimized.

All studies were carried out in accordance with relevant standards after obtaining the approval of the Institutional Review Board (IRB number: 1376) at Wonkwang University Hospital. All patients received a clear and sufficient explanation of the study purpose, contents, methods, possible PF side effects, and compensation standards and then provided signed informed consent.

### 2.3. Plant Materials and Extract Preparation

PF was purchased from Yuil Pharm, a qualified oriental drug store, located in Seoul Kyungdong Market. The purchased product was purified at the Korean Medicine Hospital of Wonkwang University and was used to fill capsules after manufacturing with powder at Hanpoong Pharm. PF (2 kg) was added to 30 L water and boiled at 100°C for 2 hours. The liquid extract was filtered and dried in a stream of hot air to obtain the powder extract followed by rotary evaporation. Recovery was approximately 20%. The powder extract was produced in granules and PF volume per capsule was approximately 400 mg. Each patient consumed 4 capsules daily, with 2 capsules taken prior to breakfast and the other 2 taken prior to lunch, each with 200 mL water. The total daily dose was 1600 mg, which was the lowest dose on the IRB recommendation.

### 2.4. Evaluation of Defecation

Survey of defecation consisted of constipation scores and morphological features of stools. The constipation score was calculated as the sum of the score of each item, ranging within 0~3 points, by adapting Rome Criteria II [10] for six items. The Bristol Stool Form Scale was used to clarify the morphological features of the stools. The scale can visually identify stool shapes and classify stool types from 1 to 7 based on shape and hardness [11].

### 2.5. Stool Retention Score Using Plain Abdominal Radiography

Stool retention score was estimated using plain abdominal radiography. The radiography images excluded all patient information. The specialists in the Radiology Department evaluated the degree of defecation retention using the Leech method [12]. In evaluating the faecal loading, each segment was given a score from 0 to 5, where 0 indicates no faeces visible, 1 indicates scanty faeces visible, 2 indicates mild faecal loading, 3 indicates moderate faecal loading, 4 indicates severe faecal loading, and 5 indicates severe faecal loading with bowel dilation [12].

### 2.6. Colon Transit Time

To evaluate the colonic mobility, colon transit time was measured. Plain abdominal radiography was carried out 4 days after administering a capsule of Kolomark (MI Tech, Seoul, Korea) for 3 days every morning at 9 AM. The capsule contains 20 rings of radiation nonpenetrating marker. As reported by Arhan et al. [13], in abdominal radiographs, the colon is segmented into 3 sections (right colon, left colon, and rectal colon) to measure the colon transit time in segments. Segmental and whole colon transit times were calculated by the number of observed markers.

### 2.7. Incidence and Severity of Side Effects

The following items were evaluated every day to estimate side effects during PF administration: soft stools, diarrhea, stomachache, abdominal displeasure, headache, vomiting, dizziness, and other effects. The degrees of side effects were scored as 0, 1, 2, and 3 points for no, light, medium, and severe side effects, respectively. The correlations of side effects with PF administration were assessed as “yes,” “possible,” and “no”; hematologic and chemistry tests were conducted every week to check the side effects that come with administration of PF.

### 2.8. Statistical Analysis

Statistical analysis was performed using the Statistical Package for the Social Sciences version 11.0 (SPSS, Chicago, IL). The differences in constipation scores, Bristol Stool Form Scale, stool retention score using plain abdominal radiograph, and colon transit times were compared before and after PF administration by paired *t*-test. Statistical significance level was *p* < 0.05.

## 3. Results

### 3.1. General Characteristics of Subjects

All 25 patients who were selected as final subjects were given PF with no deviations from the dosing schedule. The study was completed by evaluating surveys, conducting plain abdominal radiography, and measuring colon transit time before and after PF administration. Five of the 6 patients who failed during the study had arbitrarily halted the administration or could not maintain dosing frequency or time. One patient complained of a medium soft stool and requested to stop PF use.

The 25 subjects comprised 22 males and 3 females from the range of 18 to 88 years of age (average age 50.9 ± 17.3 years). The period from SCI to study participation was 5.3 ± 6.0 months on average. According to the American Spinal Cord Injury Association Impairment Scale (AIS), 5 people were classified as A, 5 people were classified as B, 4 people were classified as C, and 11 people were classified as D. Fourteen subjects had cervical SCI and 11 had thoracolumbar SCI (Table 1).

### 3.2. Evaluation of Bowel Pattern

Prior to administering PF, the constipation score averaged 4.60 ± 3.35 points ranging from 1 to 15 points. The score after PF administration averaged 3.48 ± 2.42 points ranging from 1 to 9 points. The decreased postadministration score was significant (*p* < 0.05). Morphologically, the average stool score before PF administration was 3.52 ± 1.33 points ranging from 1 to 5 points. The postadministration score averaged 4.32 ± 1.44 points ranging from 1 to 6 points. The pre- to postadministration difference was significant (*p* < 0.05, Table 2).

### 3.3. Stool Retention Score Using Plain Abdominal Radiography

Stool retention score before and after PF administration was 7.25 ± 1.60 and 6.46 ± 1.53 points in the whole colon, respectively. The postadministration decrease was significant (*p* < 0.05). The score in each segment before and after PF administration was 2.45 ± 0.61 and 1.90 ± 0.64 points in the right colon, 2.30 ± 0.86 and 2.20 ± 0.69 points in the left colon, and 1.90 ± 0.85 and 1.40 ± 0.8 points in the rectal colon, respectively. The right colon and the rectal colon exhibited statistical significance (both *p* < 0.05, Table 3).

### 3.4. Colon Transit Time

Colon transit time before and after PF administration was 57.41 ± 20.7 and 41.2 ± 25.5 hours for whole colon transit time, respectively. The postadministration decrease was significant (*p* < 0.05). Transit time for each segment before and after PF administration was 14.4 ± 16.2 and 10.1 ± 12.1 hours in the right colon, 21.8 ± 12.3 and 14.8 ± 11.8 hours in the left colon, and 20.8 ± 12.1 and 16.3 ± 14.2 hours in the rectal colon, respectively. The right and the left colon exhibited statistical significance (both *p* < 0.05, Table 3).

### 3.5. Incidence and Severity of Side Effects

Side effects after administering PF were observed in 9 out of the 25 (36.0%) patients, including 2 patients with soft stools and 7 patients with diarrhea. Two of the patients who complained of diarrhea were diagnosed as pseudomembranous colitis, which is considered to be unrelated to PF administration. The final occurrence for side effects was evaluated in 7 patients (28.0%). Side effects were rated as light in 3 patients and medium in 2 patients. After study termination, patients were monitored for 3 months to identify other side effects. One patient was diagnosed as cardiac arrhythmia thrombus 43 days after study termination. This was considered unrelated to PF administration (Table 4). In the hematologic and chemistry test, there were no shown side effects within the clinical trial.

## 4. Discussion

PF is an immature fruit of* Poncirus trifoliata* (L.) Raf. and* Citrus aurantium* var.* daidai* Mak., belonging to Rutaceae. PF is a traditional medicine used for various gastrointestinal diseases in Southeast Asia including Korea. The Korean pharmacopoeia regulates PF as an immature fruit of* Poncirus trifoliata* (L.) Raf., with a diameter of 1~2 cm, and its recommended single dosage is 2~75 g [6].

PF includes over 50 phytochemicals including poncirin, limonene, synephrine, hesperidin, neohesperidin, auraptene, and imperatorin [6]. Various ingredients of PF are used to treat many different diseases and relieve symptoms. The various biological effects include induction of apoptosis [14], antiplatelet [15], antibacterial [16], and antiallergic [17] activities. PF has been widely used for the treatment of gastrointestinal (GI) disorders related to abnormal GI motility and gastric secretion, especially for traditional medicines used for constipation in Korea and Southeast Asia. Particularly, the aqueous extract of PF (PF-W) is used for the treatment of digestive dysfunctions that include constipation, diarrhea, and dyspepsia [6].

Administration of PF promoted peristaltic movements of the small intestine and shortened the colon transit time in experimental animals and normal adults [7, 8]. In addition, defecation weight and number reportedly increased after PF administration in a mouse SCI model which was followed by increasing spontaneous contraction [18]. Authors of [18] concluded that PF administration is efficient in improving colon motility. However, data from humans are scant.

Presently, a 2-week administration schedule of PF significantly improved the constipation score and the morphological features of stools, improved the degree of defecation retention, and showed a decrease in whole colon transit time using plain abdominal radiography. This result is identical to a previous report [7, 9] that the oral administration of PF aqueous extract can promote the transit of intestinal contents. This result is meaningful in that PF administration led to an increase of colon motility even in SCI patients with neurogenic bowels, suffering from a delay in colon motility. The finding warrants further study of the influence of PF effects on primary or secondary constipation caused by various diseases.

The prokinetic effects of PF-W are well known but the mechanism of action is still unclear. With the influence of PF on GI tract motility, the action of serotonin receptor subtype 4 (5-HT4R) has been implicated in the prokinetic mechanism of PF-W [19]. Also, the action of PT hexane extract could be caused by activation of acetylcholinergic M2 and M3 receptors [20]. The methanol extract of PF modulates pacemaker potentials through 5-HT3 and 5-HT4 receptor mediated pathways via external Na^+^ and Ca^2+^ influx and via Ca^2+^ release from internal stores in a mitogen-activated protein (MAP) kinase dependent manner [21]. In addition, PF-W contains components, which can activate the ghrelin receptor that is responsible for the strong prokinetic activity of PF-W [22]. Considering the change of muscarinic (M) receptors in the large intestine, such as an increase in the density of all muscarinic (M) receptors and the change in receptor subtype from M3 to M2 [23] in bladder or large intestine of mouse model, it could be explained that PF promotes prokinetic activity through the 5-HT4R-mediated pathway.

The influence of PF on the GI tract is multifactorial and there may be a difference in the effect based on the type of extract, whether it is aqueous solution, hexane, or methanol. Aqueous extract of PF has been amply linked with prokinetic activity. Several reports described that only hexane extract of PT can dose-dependently increase the low frequency contraction of longitudinal muscle in distal colon strips [20] and that methanol extract of PT produces prokinetic activity through the mitogen-activated protein kinase pathway. This study used an aqueous extract, so it might require additional studies based on the various extraction methods.

The recommended general over-the-counter dosage of PF is 2~75 g. PF-W was reportedly nontoxic even at a dose of 5 g/kg when orally given to mice [7]. But the most efficient dosage and administration method according to the type of GI disorder are still not clear, so the standard is not yet established.

In this study, daily dosages of 1600 mg were administered considering the stability of patients, and the lowest over-the-counter dosage was represented. PF side effects related to GI tract include soft stools, diarrhea, and stomachache. However, the severity and occurrence rates of side effects in relation to dosage have not been reported before. In this study, PF side effects were observed in 7 out of 25 patients (28%). Side effects included soft stools for 2 patients and diarrhea for 5 patients, with the severity being ranked as light in 5 people and medium in 2 people. One patient who had complained of a medium soft stool and asked to halt PF administration showed improved symptoms 2 days after halting PF use. Further study could be instructive in deducing the best administration method and dosing of PF.

Generally, the evaluation of neurogenic bowel in patients with SCI depends on subjective symptoms, such as bowel frequency. However, previous reports show patient recall of bowel habits is sometimes inaccurate [24], so objective evaluation methods such as plain abdominal radiography and colon transit time are recommended [25]. Objective means of evaluation, such as constipation score, plain abdominal radiography, and colon transit time using Rome Criteria II used in the previous study [26], were presently adapted to estimate the effect of PF. A significant difference in postadministration was observed in constipation score, stool retention score, and colon transit time. Statistical significance was found in the right and the left colon transit time, as well as the whole colon transit time before and after PF administration. This might reflect that all subjects were inpatients and bowel managements, such as the use of laxatives, enemas, suppositories, and digital rectal stimulation, affected primarily the rectosigmoid colon. Thus, there may be an error in measuring the rectosigmoid colon transit time.

For the clinical application of PF for constipation in the future, the mechanism should be identified to promote GI tract motility and there should be standardization according to extraction methods. In addition, the administration amount should be standardized, which may enhance GI tract movements.

In summary, PF could improve defecation and side effects even in SCI patients with neurogenic bowels. There can be further exploration of the difference in effects based on various extraction methods as well as further exploration relating PF dosing and its side effects.

## 5. Conclusions

In order to evaluate the effect of PF on the colon motility of SCI patients with neurogenic bowel, this study estimated the change of defecation patterns by using constipation score, Bristol Stool Form Scale, stool retention degree through plain abdominal radiography, and colon transit time. Significant improvements after 14 days of PF administration were observed in bowel habits, stool retention, and colon transit time. The data showed PF enhances colon motility and improves constipation symptoms.

## Figures and Tables

**Table 1:** General characteristics of patients with spinal cord injury.

Demographic factor	Value
Total number of cases	25

Mean age (years)	50.9 ± 17.3

Sex (male/female)	22/3

Duration of injury (months)	5.3 ± 6.0

Cause of injury	
Traumatic	19
Transverse myelitis	1
Other	5

Level of injury (ASIA scale)	
Cervical (A/B/C/D)	14 (2/2/3/7)
Thoracolumbar (A/B/C/D)	11 (3/3/1/4)

Values are number or mean ± standard deviation.

ASIA, American Spinal Cord Injury Association.

**Table 2:** The results of constipation score and Bristol Stool Form Scale between pretreatment and posttreatment.

	Pretreatment	Posttreatment	*p* value
Constipation score	4.60 ± 3.35	3.48 ± 2.42	0.04^*∗*^
Bristol Stool Form Scale	3.52 ± 1.33	4.32 ± 1.44	0.03^*∗*^

*∗* denotes significant difference in the PF treatment before and after (^*∗*^ *p* < 0.05).

**Table 3:** The results of colon transit time and stool retention score in the PF treatment before and after.

	Before	After

CTT		
Right colon	14.4 ± 16.2	10.1 ± 12.1^*∗*^
Left colon	21.8 ± 12.3	14.8 ± 11.8^*∗*^
Rectosigmoid colon	20.8 ± 12.1	16.3 ± 14.2
Total	57.4 ± 20.7	41.2 ± 25.5^*∗*^

Stool retention score		
Right colon	2.45 ± 0.61	1.90 ± 0.64^*∗*^
Left colon	2.30 ± 0.86	2.20 ± 0.69
Rectosigmoid colon	1.90 ± 0.85	1.40 ± 0.88^*∗*^
Total	7.25 ± 1.60	6.46 ± 1.53^*∗*^

CTT: colon transit time, each value expressed as mean ± standard deviation.

*∗* denotes significant difference in the PF treatment before and after (^*∗*^ *p* < 0.05).

**Table 4:** The type, number, and severity of adverse event.

Adverse event	Number	Severity
Loose stool	2	Mild: 2

Diarrhea	5	Mild: 3 Moderate: 2
